# Extracellular Volume Fraction Based on Cardiac Magnetic Resonance T1 Mapping: An Effective Way to Evaluate Cardiac Injury Caused by Cardiac Amyloidosis in Patients with Multiple Myeloma

**DOI:** 10.1155/2022/3094933

**Published:** 2022-08-13

**Authors:** Minghui Liu, Liang Shao, Zhaoxia Yang, Qian Wang, Balu Wu, Xiaoyan Liu, Yalan Yu, Tingting Huang, Meixing Wang, Yong He, Guohong Liu, Fuling Zhou

**Affiliations:** ^1^Department of Hematology, Zhongnan Hospital of Wuhan University, Wuhan 430071, China; ^2^Department of Radiology, Zhongnan Hospital of Wuhan University, Wuhan 430071, China; ^3^Department of Nuclear Medicine, Zhongnan Hospital of Wuhan University, Wuhan 430071, China

## Abstract

Multiple myeloma (MM) is a hematological malignancy of plasma cell origin. Cardiac amyloidosis (CA) is a common form of heart damage caused by MM and is associated with a poor prognosis. This study was a prospective cohort study and was aimed at evaluating the clinical predictive value of extracellular volume fraction (ECV) based on cardiovascular magnetic resonance (CMR) T1 mapping for cardiac amyloidosis and cardiac dysfunction in MM patients. Fifty-one newly diagnosed MM patients in Zhongnan Hospital of Wuhan University were enrolled in the study. A total of 19 patients (19/51; 37.25%) developed CA. The basal ECV of CA group was significantly higher than that of the non-CA group (*p* < 0.01). Multivariate logistic regression analysis showed that basal ECV (OR = 1.551, 95% CI 1.084-2.219, *p* < 0.05) and LDH1 level (OR = 1.150, 95% CI 1.010-1.310, *p* < 0.05) were two independent risk factors for CA. Further study demonstrated that basal ECV in the heart failure group was significantly higher than that of the nonheart failure group (*p* < 0.01). Notably, ROC curve showed that basal ECV had a good predictive value for CA and heart failure, with AUC of 0.911 and 0.893 (all *p* < 0.01), and the best cutoff values of 38.35 and 37.45, respectively. Taken together, basal ECV is a good predictor of CA and heart failure for MM patients.

## 1. Introduction

Symptomatic light chain (AL) amyloidosis occurs in 15%~30% of multiple myeloma (MM) patients and becomes more severe when the disease progresses. Cardiac amyloidosis (CA) is a condition in which primary or secondary factors cause the deposition of amyloid protein in the heart muscle, causing abnormalities in the structure and function of the heart [1–3].

Endomyocardial biopsy (EMB) is the gold standard for the diagnosis of CA. However, it is invasive and needs advanced technical skills to obtain cardiac tissues [4]. BNP, pro-BNP, troponin, and electrocardiogram are general markers for detecting myocardial injury, but their specificity is limited. Alternatively, echocardiography is a crucial noninvasive method for the diagnosis of CA secondary to MM. At the initial stage of the disease, the main symptoms were mild to moderate diastolic function limitation, but the left ventricular ejection fraction was often greater than 50% [5]. Once CA is diagnosed by echocardiography, it is already very severe and the prognosis is very poor [6]. Therefore, an imaging method with a high sensitivity and specificity is urgently needed for detecting early cardiac injury in MM patients with amyloidosis, as well as indicators of the extent of impairment of cardiac function caused by amyloidosis.

Cardiac magnetic resonance (CMR) can comprehensively evaluate the size of the heart cavity, the thickness of the atrioventricular wall, myocardial movement, and the state of cardiac function. Although late gadolinium enhancement (LGE) is useful in the early detection of CA [7], it is not a quantitative indicator. Extracellular volume fraction (ECV) is the fractional volume of water outside of cells in relation to the volume of water inside of cells in myocardial tissue. An increased ECV value indicates the presence of excessive collagen deposition or fibrosis, such as in amyloidosis or myocardial infarction. Measurements of ECV based on contrast enhanced CMR T1 mapping showed promise in detecting CA quantitatively with highest reproducibility and insight into the severity of amyloid deposition [8]. In this study, we investigated the clinical predictive value of ECV for CA and cardiac dysfunction in patients with MM.

## 2. Patients and Methods

### 2.1. Patients

In the present study, a total of 51 newly diagnosed multiple myeloma (MM) patients were enrolled in the Department of Hematology, Zhongnan Hospital of Wuhan University, during the period from May 2019 to June 2021. Exclusion criteria are as follows: (1) patients with chronic kidney disease, kidney transplantation, or single kidney and (2) patients with a history of coronary heart disease, anemic heart disease, cardiac dysfunction, or cardiac amyloidosis due to other diseases. This study was approved by the Ethics Committee of Zhongnan Hospital of Wuhan University (Registered on NIH Clinical Trials: NCT05034146).

According to the diagnostic criteria of AL amyloidosis in immunoglobulin light chain amyloidosis: 2020 update on diagnosis, prognosis, and treatment, the patients were divided into groups with and without cardiac amyloidosis. The differences between the two groups were compared. Diagnoses of cardiac amyloidosis are as follows: (1) the patient has corresponding clinical symptoms, (2) abnormal light chain examination, (3) amyloid deposits confirmed by bone marrow biopsy, and (4) CMR results which support amyloidosis [9]. According to the diagnostic criteria, they were divided into groups without heart failure and groups with heart failure. Diagnoses of no heart failure group are as follows: (1) BNP < 35 pg/ml and NT − proBNP < 125 pg/ml and (2) ECG and chest radiography showed no obvious abnormality. The rest were divided into groups with heart failure [10].

### 2.2. Laboratory Parameters

Bone marrow biopsy was conducted and viewed in fluorescence microscope to confirm amyloid deposits. Biomarkers for detecting myocardial injury such as *β*2-MG, LDH1, hypersensitive TNI, pro-BNP, and LVEF were measured from the blood sample of patients with multiple myeloma.

### 2.3. CMR Examination

All participants underwent standard CMR examinations on a 3.0 T MR scanner (Prisma, Siemens Healthcare, Erlangen, Germany) equipped with an 18-channel phased-array body coil combined with 12 channels in the supine position. Pre- and postcontrast T1 mapping was acquired using a modified Look-Locker inversion recovery (MOLLI) sequence with 5(3)3 and 4(1)3(1)2 schemes, respectively. T1 mapping images were collected at the base, midventricle, and apex on the short-axis left ventricle (LV). LGE imaging was performed by using a phase-sensitive inversion-recovery (PSIR) gradient-echo pulse sequence. LGE images were acquired 10 to 15 min after administration of contrast agent (0.2 mmol/kg Dotarem, Paris, France), covering three long-axis planes (2-3-4 chamber) and short-axis slices from the atrioventricular ring to the LV apex.

### 2.4. ECV Quantification Based on Mapping of CMR

All CMR image analyses were performed with an offline commercial software (cvi42, v.5.13.5; Circle Cardiovascular Imaging, Calgary, Canada). Native T1 and post-T1 values were measured by drawing a region of interest on the septum myocardium of the basal, midventricle, and apex short-axis slice images (this was done blinded to the LGE images). ECV was calculated by the following equation: ECV = (1/T1myo post − 1/T1 myo pre) × (1 − Hct)/(1/T1blood post − 1/T1 blood pre). Hematocrit was obtained on the same day of the CMR scans. The LGE pattern was classified into characteristic for CA and negative for CA. According to the nomenclature and segmentation of the left ventricular myocardium (the cardiac segmentation model) of the American Heart Association (AHA), in the long axis, the left ventricle is divided into equal thirds named the basal, mid, and apical thirds. The tip of the apex forms a separate final segment. When these thirds are viewed in short axis, they form rings that are numbered counterclockwise which can be further divided into equal sectors: the basal third with six 60° sectors, the mid third with six 60° sectors, and the apical third with four 90° sectors. The average ECV of the equal thirds are named basal ECV, mid ECV, and apical ECV.

### 2.5. Statistical Analysis

SPSS 26.0 statistical software was used for data analysis. The measurement data are normally distributed by using the Shapiro-Wilk test. Those conforming to the normal distribution are represented by mean ± SD. Independent sample *T* test was used for intergroup comparison. The ones that do not fit the normal distribution are represented by M (1/4, 3/4). The Wilcoxon rank sum test was used for comparison between groups. The counting data are expressed in percentage numbers. *χ*^2^ or Fisher's exact test was used for comparison between groups. Multivariate logistic regression analysis was performed to find independent risk factors for cardiac amyloidosis and neofunctional dysfunction after excluding indicators that caused significant collinearity. The Spearman method was used to analyze data correlation. The receiver operating characteristic (ROC) curve was plotted, and the area under curve (AUC) was calculated to evaluate the predictive effectiveness of ECV. All tests were bilateral tests, and *p* < 0.05 was considered statistically significant.

## 3. Results

### 3.1. Clinical Characteristics of Patients in Cardiac Amyloidosis and Noncardiac Amyloidosis Groups

**Figure 1:**
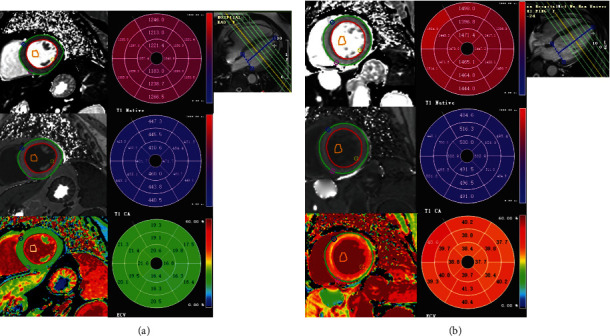
CMR pre- and postcontrast T1 mapping and calculated ECV mapping of representative MM patients (a) without or (b) with CA.

**Figure 2:**
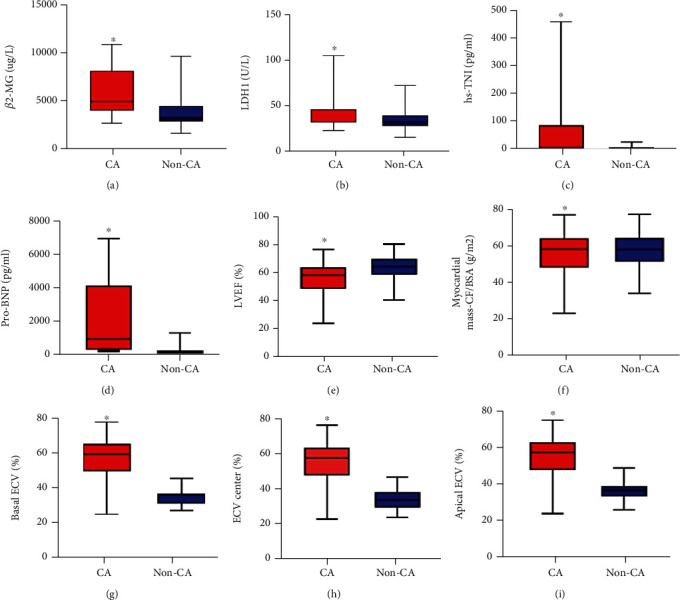
The comparison of biochemical parameters between patients with or without CA. (a) *β*2-microglobulin (*β*2-MG) (CA, *n* = 19; non-CA, *n* = 32). (b) Lactate dehydrogenase 1 (LDH1) (CA, *n* = 19; non-CA, *n* = 32). (c) High-Sensitivity Troponin I (hs-TNI) (CA, *n* = 19; non-CA, *n* = 32). (d) Pro-brain natural peptide (pro-BNP) (CA, *n* = 19; non-CA, *n* = 32). (e) Left ventricular ejection fraction (LVEF) (CA, *n* = 19; non-CA, *n* = 32). (f) Myocardial mass (CF/BAS) (CA, *n* = 19; non-CA, *n* = 32). (g) Basal ECV (CA, *n* = 19; non-CA, *n* = 32). (h) Mid ECV (CA, *n* = 19; non-CA, *n* = 32). (i) Apical ECV (CA, *n* = 19; non-CA, *n* = 32).

The clinical characteristics of the participants are shown in Table 1. Nineteen MM patients developed CA, with an incidence of 37.25% (19/51). Deposition of amyloid in bone marrow was confirmed by fluorescence microscope and bone marrow biopsy. Examples of CMR pre- and postcontrast T1 mapping and calculated ECV mapping of representative MM patients with or without CA are shown in Supplementary Figure 1. Examples of CMR pre (native T1) and postcontrast (CA T1) T1 mapping and calculated ECV mapping of patients with multiple myeloma and without or with amyloidosis are shown in the polar map in Figures 1(a) and 1(b). The green and red lines on the T1/ECV mapping image outlined the epi and endo septum myocardium, while the area inside the orange line represents the blood pool. The T1 mapping of pre- and postcontrast injection were shown in gray scale, while in the polar map (defined by AHA as described above), blue and red color bars represent low and high T1 value, respectively. For the ECV map, the color bar changes from blue to green, yellow, and red with increased ECV values. Basal ECV, mid ECV, and apical ECV of patients with multiple myeloma were extracted and analyzed. We can see from Figure 1 that patient of multiple myeloma with amyloidosis showed an obviously higher ECV than patient without amyloidosis. Basal ECV, mid ECV, and apical ECV of patients with multiple myeloma were extracted and analyzed. In order to present the clinical characteristics of CA patients, we collected and compared the laboratory data between CA and non-CA patients. As shown in Figure 2, laboratory parameters such as *β*2-MG, LDH1, hypersensitive TNI, pro-BNP, basal ECV, apical ECV, and mid ECV were significantly higher in the CA group than those in the non-CA group (all *p* < 0.05), while LVEF value was lower than that in the non-CA group (*p* < 0.05). There was no significant difference in Durie-Salmon (DS) staging and International Staging System (ISS) staging between the two groups (all *p* > 0.05) (Supplementary Table 1).

### 3.2. Comparison of Laboratory Parameters between the Heart Failure and Nonheart Failure Groups

**Figure 3:**
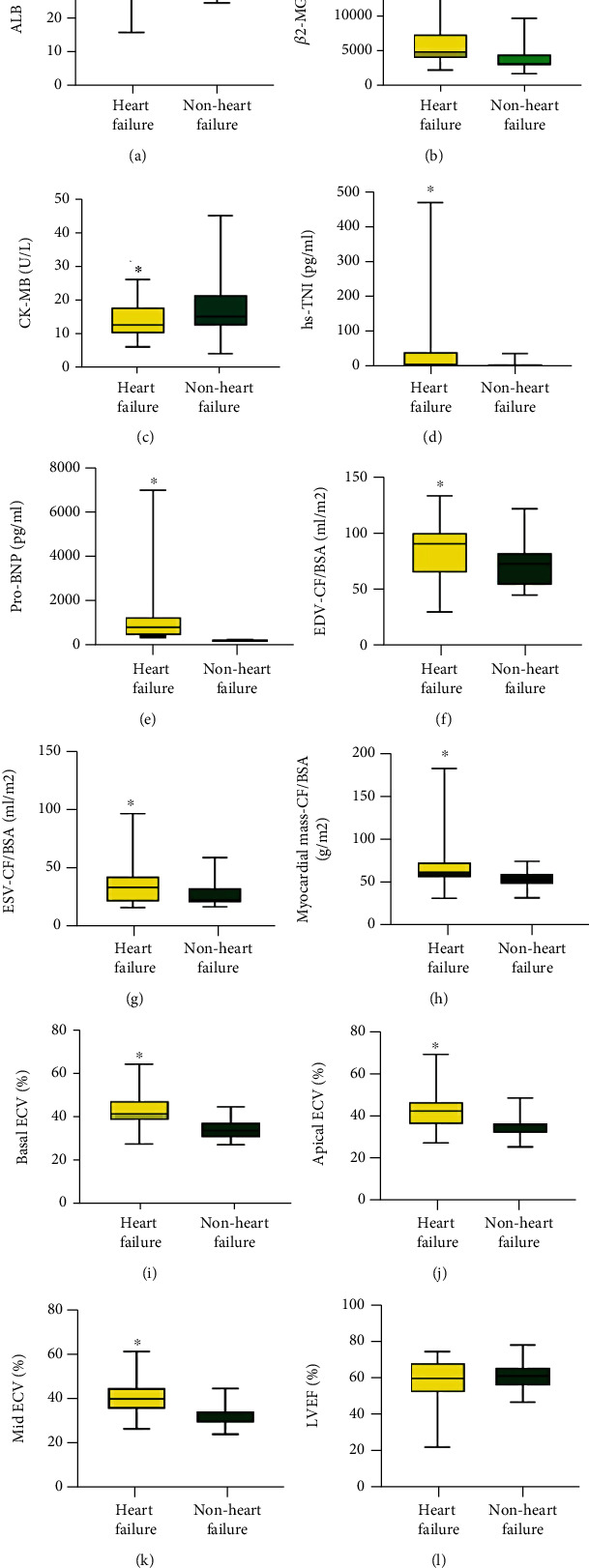
The comparison of biochemical parameters between patients with or without heart failure. (a) ALB (heart failure, *n* = 30; nonheart failure, *n* = 21). (b) *β*2-MG (heart failure, *n* = 30; nonheart failure, *n* = 21). (c) Creatine Kinase-MB (CK-MB) (heart failure, *n* = 30; nonheart failure, *n* = 21). (d) hs-TNI (heart failure, *n* = 30; nonheart failure, *n* = 21). (e) pro-BNP (heart failure, *n* = 30; nonheart failure, *n* = 21). (f) End-diastolic volume (EDV)-CF/BAS (heart failure, *n* = 30; nonheart failure, *n* = 21). (g) End-systolic volume (ESV)-CF/BAS (heart failure, *n* = 30; nonheart failure, *n* = 21). (h) Myocardial mass (CF/BAS) (heart failure, *n* = 30; nonheart failure, *n* = 21). (i) Basal ECV (heart failure, *n* = 30; nonheart failure, *n* = 21). (j) Apical ECV (heart failure, *n* = 30; nonheart failure, *n* = 21). (k) Mid ECV (heart failure, *n* = 30; nonheart failure, *n* = 21). (l) Left ventricular ejection fraction (LVEF) (heart failure, *n* = 30; nonheart failure, *n* = 21).

According to the 2021 ESC Guidelines for the diagnosis and treatment of acute and chronic heart failure, thirty MM patients met the diagnosis criteria of acute or chronic heart failure. Compared with the nonheart failure group, the heart failure group exhibited higher levels of *β*2-MG, hypersensitive TNI, pro-BNP, EDV (CF/BSA), ESV (CF/BSA), myocardial mass (CF/BSA), basal ECV, apical ECV, and mid ECV (all *p* < 0.05). ALB and CK-MB were lower than that of the nonheart failure group (*p* < 0.05) (Figure 3). There was no statistical significance in other indicators between the two groups (all *p* > 0.05) (Supplementary Table 2).

### 3.3. Analysis of Risk Factors for CA

**Figure 4:**
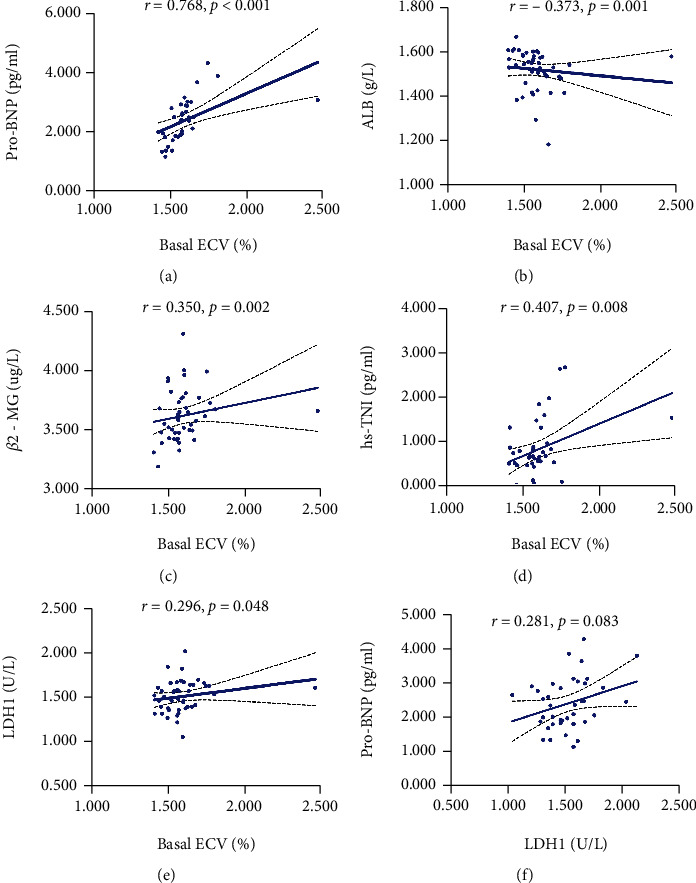
The correlation between basal ECV and other laboratory parameters in 51 MM patients. (a) pro-BNP, (b) ALB, (c) *β*2-MG, (d) hs-TNI, and (e) LDH1. The correlated analysis between pro-BNP and LDH1 was shown (f). Spearman's correlation analysis and equation of residuals plots were shown.

Next, we further determined the risk factors for CA in MM patients. Because there was collinearity between basal ECV, apical ECV apex, and mid ECV, we used basal ECV, *β*2-MG, LDH1, hypersensitive TNI, pro-BNP, and LVEF as independent variables to construct the regression model. Multivariate logistic regression analysis showed that basal ECV (OR = 1.551, 95% CI 1.084-2.219, *p* = 0.016) and LDH1 (OR = 1.150, 95% CI 1.010-1.310, *p* = 0.036) were independent risk factors for CA (Table 2). The correlation analysis showed that basal ECV base was positively correlated with pro-BNP (*r* = 0.768, *p* < 0.001), *β*2-MG (*r* = 0.350, *p* = 0.002), hs-TNI (*r* = 0.407, *p* = 0.008), and LDH1 (*r* = 0.296, *p* = 0.048) but was negatively correlated with ALB (*r* = −0.373, *p* = 0.001) (Figure 4).

### 3.4. Analysis of Risk Factors for Heart Failure

Next, we tried to determine the risk factors for heart failure in MM patients. In this regression mode, heart failure was taken as a dependent variable. LVEF, basal ECV, LDH1, CK-MB, EDV (CF/BSA), and ESV (CF/BSA) were used as independent variables. Multivariate logistic regression analysis showed that basal ECV (OR = 1.329, 95% CI 1.080-1.635, *p* = 0.007) was an independent risk factor for heart failure (Table 3).

### 3.5. The Predictive Value of Basal ECV in CA and Heart Failure

**Figure 5:**
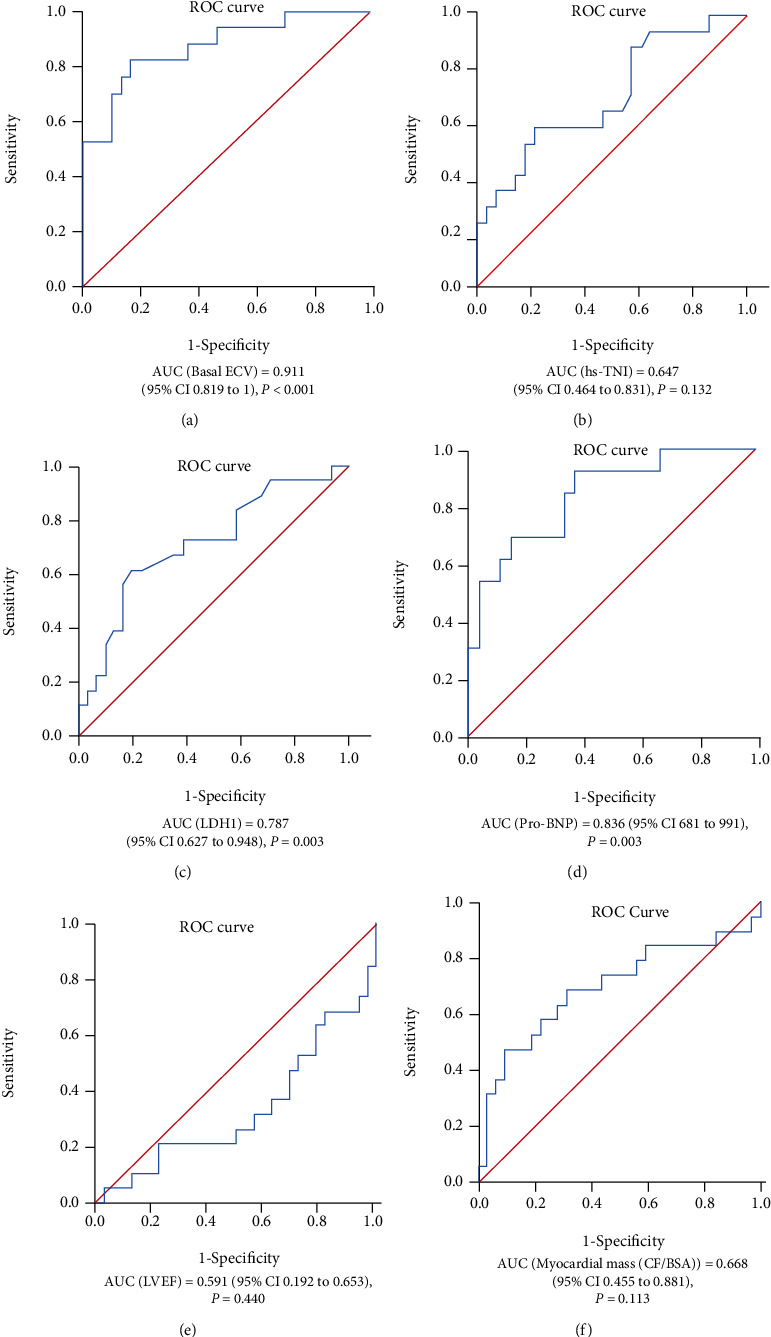
ROC curve for predicting CA by (a) basal ECV, (b) hs-TNI, (c) LDH1, (d) pro-BNP, (e) LVEF, and (f) myocardial mass (CF/BSA).

Next, we tested the sensitivities of basal ECV, hs-TNI, LDH1, pro-BNP, LVEF, and ESV (CF/BSA) alone in the prediction of the risk of CA. As shown in Figure 5, the AUC from ROC analysis demonstrated that the predictive value of basal ECV for CA is 0.911 (CI, 0.819 to 1). The best predictive cutoff value of basal ECV for the primary outcome was 38.35, with a sensitivity of 92.9% and a specificity of 80%. Patients with a basal ECV value > 38.35 showed a high risk for CA and might need timely therapeutic intervention. The following AUCs were pro-BNP (0.836) > LDH1 (0.787) > *β*2-MG (0.682) > myocardial mass (CF/BSA) (0.668) > hs-TNI (0.647) (Supplementary Table 3).

**Figure 6:**
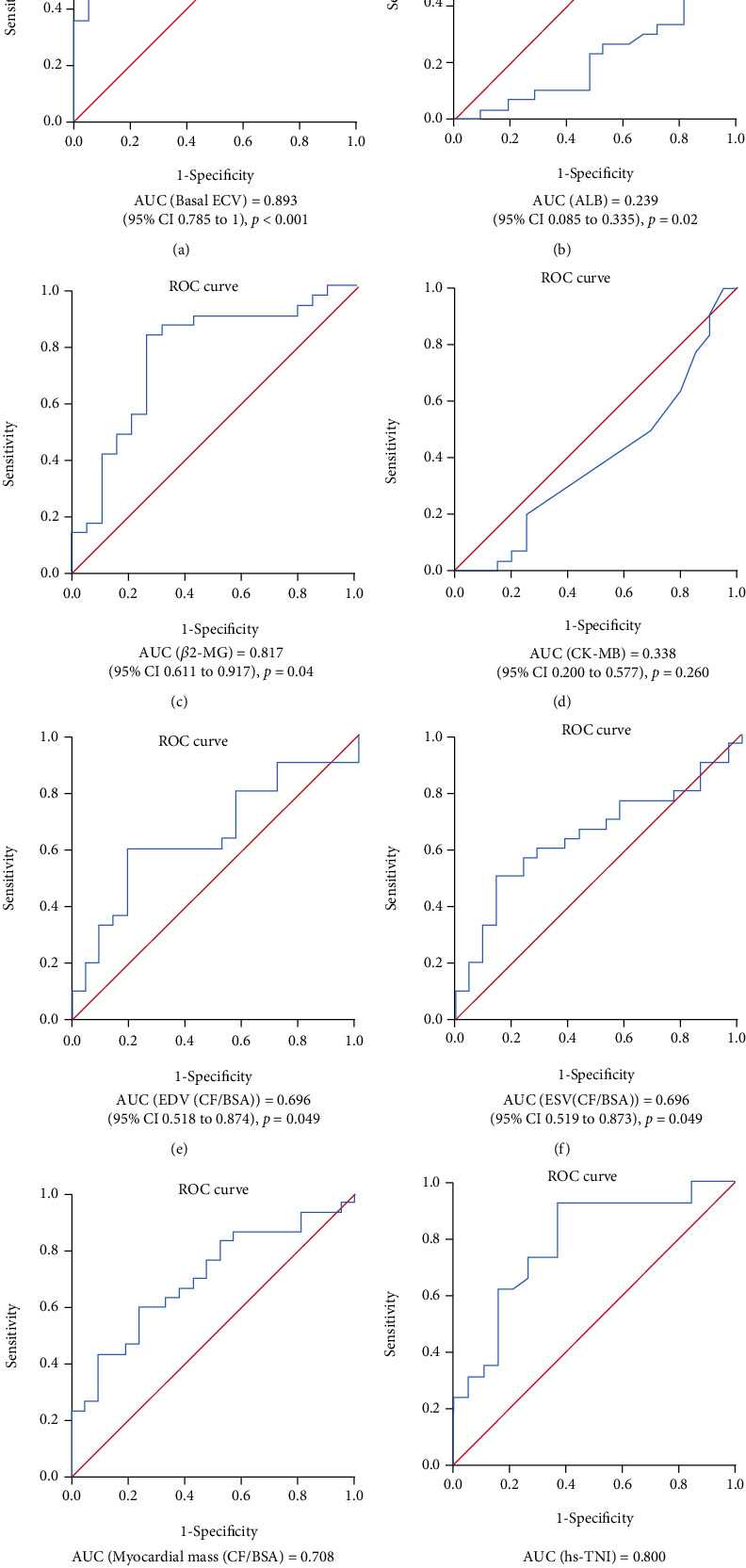
ROC curve for predicting heart failure by (a) basal ECV, (b) ALB, (c) *β*2-MG, (d) CK-MB, (e) EDV (CF/BSA), (f) ESV (CF/BSA), (g) myocardial mass (CF/BSA), and (h) hs-TNI.

Finally, as shown in Figure 6, we determined the sensitivities of basal ECV, hs-TNI, LDH1, pro-BNP, LVEF, and ESV (CF/BSA) alone in the prediction of the risk of heart failure. The AUC from ROC analysis demonstrated that the predictive value of basal ECV for heart failure is 0.893 (CI, 0.785 to 1). The best predictive cutoff value of basal ECV for the primary outcome was 37.45, with a sensitivity of 70.4% and a specificity of 93.7%. Patients with a basal ECV value > 37.45 showed a high risk for heart failure and might need timely therapeutic intervention. The following AUCs were *β*2-MG (0.817) > hs-TNI (0.8) > myocardial mass (CF/BSA) (0.708) > EDV (CF/BSA) (0.696) = ESV (CF/BSA) (0.696) (Supplementary Table 4).

## 4. Discussion

The kappa or lambda-type single-cloned light chain produced by marrow slurry cell tumors is the main cause of AL amyloidosis. Amyloidosis can occur in any immunoglobulin-secreting B-cell tumor, including MM, macroglobulinemia, CLL, and nonlymphocytic lymphoma [11]. The focus of our discussion is CA caused by AL amyloidosis. CA is a severe manifestation of systemic amyloidosis, manifested as restrictive cardiomyopathy. Amyloid can be deposited in any part of the heart, including the heart muscle, endocardium, valves, endothelium, and pericardium.

The toxicity of light chain and amyloid deposits leads to extensive damage to cardiac tissues, ultimately leading to cardiac dysfunction. Other mechanisms include disturbance of cell membranes by amyloid fibrils, cytotoxicity caused by fibrils growth, and formation of soluble light chain oligomers by amyloid fibrils. In addition, the light chain of soluble amyloid protein induces cell apoptosis. Amyloid light chains induce MAPK signaling, leading to increased production of reactive oxygen species (ROS), impaired calcium homeostasis, cellular dysfunction, and death of adult cardiomyocytes [12].

The sensitivity and negative predictive values of basal ECV on CMR T1 mapping for the diagnosis of CA in AL were both 100%, while the specificity and positive predictive values were 80% and 81%, respectively [7]. LGE of CMR is an independent risk factor for mortality in patients with AL CA and has a high prognostic value, but LEG still has some limitations [13].

ECV showed a promise in noninvasive diagnosis of CA [14]. ECV has a good repeatability (independent of field strength or technical differences) and provides insight into the severity of amyloid deposition [15]. Moreover, ECV is quantifiable and can be a useful parameter of therapeutic response.

The Mayo Clinic presented two prognostic models for AL amyloidosis in 2004 and 2012, respectively. In addition, there is a European model. The European and Mayo 2012 models have shown good predictive performance in recent validation studies [16]. However, both troponin and brain natriuretic peptide are susceptible to other factors besides amyloidosis, such as renal insufficiency, coronary heart disease, and anemic heart disease. Yang et al. reported a positive correlation between ECV, hs-TNI, and pro-BNP levels in patients with heart failure. It also suggests that ECV may contribute to additional heart failure risk stratification [17].

To our knowledge, this study was the first to evaluate the predictive value of basal ECV for CA and heart failure in MM patients. The results indicate that basal ECV is a good predictor of CA and heart failure in patients with MM. Basal ECV can effectively identify patients with cardiac dysfunction caused by AL amyloidosis, which is helpful for the selection of chemotherapy regimens. The advantage of the clinical application of basal ECV can avoid invasive heart biopsy.

## Figures and Tables

**Table 1:** Clinical and laboratory characteristics of participants.

Characteristics	Amyloid group	Nonamyloid group	*p*
	(*n* = 19)	(*n* = 32)	
Age	59.89 ± 9.640	61.07 ± 8.863	0.668
*Sex*			0.091
Male	7 (13.7%)	20 (39.2%)	
Female	12 (23.5%)	12 (23.5%)	
*Medical history*			
Hypertension	7 (13.7%)	9 (17.6%)	0.639
Diabetes	3 (5.9%)	2 (3.9%)	0.348
Hyperlipidemia	0 (0%)	2 (3.9%)	0.523
COPD	2 (3.9%)	3 (5.9%)	0.966
Hepatitis B	0 (0%)	3 (5.9%)	0.285
*Heavy chain type*			0.82
IgG	5 (9.8%)	8 (15.7%)	
IgA	7 (13.7%)	9 (17.6%)	
IgM	0	1 (2%)	
*Light chain type*			0.952
*λ* light chain	9 (18.8%)	15 (31.3%)	
*κ* light chain	9 (18.8%)	15 (31.3%)	
*DS staging*			0.488
1A	1 (2%)	0	
2A	2 (4%)	4 (7.8%)	
3A	12 (23.5%)	22 (43.1%)	
3B	6 (7.8%)	6 (11.8%)	
*ISS staging*			0.419
2A	10 (19.6%)	22 (43.1%)	
3A	5 (9.8%)	7 (13.7%)	
3B	4 (7.8%)	3 (5.9%)	

**Table 2:** Multivariate logistic analysis of cardiac amyloidosis.

	*β*	S.E.	Wald	OR	95% CI	*p*
Basal ECV	0.439	0.183	5.768	1.551	1.084-2.219	0.016
LDH1	0.1409	0.066	4.451	1.15	1.010-1.310	0.036

**Table 3:** Multivariate logistic analysis of heart failure.

	*β*	S.E.	Wald	OR	95% CI	*p*
Basal ECV	0.284	0.106	7.215	1.329	1.080-1.635	0.007

## Data Availability

The data and materials in the current study are available from the corresponding author on reasonable request.

## Supplementary Materials

Supplementary Materials Supplementary Table 1: the laboratory findings of participants in the amyloid group and nonamyloid group. Supplementary Table 2: the laboratory findings of participants in the heart failure group and nonheart failure group. Supplementary Table 3: predictive value of ECV in CA. Supplementary Table 4: predictive value of ECV in heart failure. Supplementary Figure 1: fluorescence microscopic photos of amyloidosis (a) and nonamyloidosis (b) in bone marrow biopsy tissue. (a) The white arrow indicates the amyloid deposits in the blood vessel wall of the bone marrow biopsy. The black arrow indicates the trabecular bone. (b) No amyloid deposits are detected.
