# Supplementary material for: Extracellular Volume Fraction Based on Cardiac Magnetic Resonance T1 Mapping: An Effective Way to Evaluate Cardiac Injury Caused by Cardiac Amyloidosis in Patients with Multiple Myeloma
Source: J Immunol Res. 2022 Aug 13;2022:3094933. doi: 10.1155/2022/3094933 (PMC9392639; doi:10.1155/2022/3094933)
Supplement: Supplementary Materials — Supplementary Table 1: the laboratory findings of participants in the amyloid group and nonamyloid group. Supplementary Table 2: the laboratory findings of participants in the heart failure group and nonheart failure group. Supplementary Table 3: predictive value of ECV in CA. Supplementary Table 4: predictive value of ECV in heart failure. Supplementary Figure 1: fluorescence microscopic photos of amyloidosis (a) and nonamyloidosis (b) in bone marrow biopsy tissue. (a) The white arrow indicates the amyloid deposits in the blood vessel wall of the bone marrow biopsy. The black arrow indicates the trabecular bone. (b) No amyloid deposits are detected. [file 3094933.f1.docx]

**Supplementary Table 1:** The laboratory findings of participants in amyloid group and non-amyloid group

| **Characteristics** | **amyloid group** | **non-amyloid group** | ***p*** |
| --- | --- | --- | --- |
|  | **(n=19)** | **(n=32)** |  |
| **Laboratory findings** | | | |
| ALB | 31.394±5.9662 | 34.482±6.028 | 0.096 |
| GLB | 31.8 | 27.45 | 0.875 |
|  | (27.702~44.398) | (29.114~44.607) |  |
| BUN | 5.13 | 5.39 | 0.566 |
|  | (4.494~7.359) | (5.137~8.463) |  |
| CREA | 73 | 72.429 | 0.919 |
|  | (65.676~91.002) | (54.770~146.233) |  |
| UA | 370.2 | 340.6 | 0.893 |
|  | (276.984~389.961) | (314.563~390.523) |  |
| CYSC | 1.35 | 1.265 | 0.543 |
|  | (1.222~1.573) | (1.218~1.622) |  |
| β2-MG | 5077.15 | 3488.4 | 0.034 |
|  | (4451.652~6987.803) | (3094.671~6348.793) |  |
| Ca | 2.152±0.246 | 2.183±0.157 | 0.606 |
| P | 1.247±0.356 | 1.236±0.273 | 0.907 |
| CK | 45 (33.57~86.10) | 42 (41.89~84.85) | 0.668 |
| CK-MB | 12 (9.82~18.73) | 15 (13.62~18.60) | 0.101 |
| LDH | 193 (176.94~26729) | 189 (168.92~231.37) | 0.578 |
| LDH1 | 41 (31.359~59.700) | 28 (25.153~33.314) | 0.014 |
| hs-TNI | 7.3 (5.865~133.978) | 4.2 (2.569~14.170) | 0.028 |
| pro-BNP | 833.5 (347.260~3406.474) | 100 (129.805~403.443) | 0.001 |
| RV5 | 1.174±0.552 | 1.318±0.057 | 0.421 |
| SV1 | 0.738±0.654 | 0.591±0.348 | 0.378 |
| QRS | 92 | 92 | 0.699 |
|  | (84.52~108.34) | (87.63~96.02) |  |
| QT | 386 | 394 | 0.482 |
|  | (286.13~433.21) | (384.62~408.77) |  |
| QTs | 436 | 435 | 0.697 |
|  | (331~487.93) | (418.91~447.49) |  |
| PR interphase | 144 | 156 | 0.312 |
|  | (100.85~173.15) | (147.71~170.87) |  |
| LVEF | 55.674±14.571 | 63.104±9.164 | 0.039 |
| EDV | 136.126±48.530 | 136.149±38.007 | 0.999 |
| ESV | 59.19 | 47.475 | 0.333 |
|  | (45.503~77.997) | (42.861~56.834) |  |
| SV | 74.376±30.994 | 86.104±27.730 | 0.188 |
| Myocardial mass | 100.285 | 94.465 | 0.424 |
|  | (86.514~139.541) | (89.171~105.933) |  |
| CO | 6.067±2.722 | 6.491±2.431 | 0.585 |
| CI | 3.825±1.632 | 3.791±1.343 | 0.94 |
| EDV (CF/BSA) | 86.068±31.504 | 79.442±19.887 | 0.385 |
| ESV(CF/BSA) | 34.335 | 24.955 | 0.115 |
|  | (9.426~116.932) | (25.156~33.034) |  |
| SV(CF/BSA) | 47.944±19.099 | 50.464±14.928 | 0.619 |
| Myocardial mass (CF/BSA) | 66.36 | 57.235 | 0.038 |
|  | (55.546~87.397) | (51.804~66.265) |  |
| basal ECV | 42.7 | 35.25 | 0 |
|  | (27.139~91.374) | (31.399~35.655) |  |
| mid ECV | 41.5 | 33.2 | 0 |
|  | (24.384~91.904) | (31.040~35.875) |  |
| apical ECV | 44.2 | 35.7 | 0 |
|  | (23.945~106.117) | (32.839~37.841) |  |

**Supplementary Table 2:** The laboratory findings of participants in heart failure group and non-heart failure group

| Characteristics | Heart failure group (n=30) | Non-heart failure group (n=21) | *p* |
| --- | --- | --- | --- |
| Laboratory findings | | | |
| ALB | 31.28±6.07 | 36.67±4.67 | 0.03 |
| GLB | 35 | 26.8 | 0.202 |
|  | (32.65~49.51) | (24.00~36.79) |  |
| BUN | 5.17 | 5.28 | 0.82 |
|  | (5.05~7.08) | (4.459~9.81) |  |
| CREA | 74.1 | 71 | 0.317 |
|  | (71.89~93.63) | (29.89~184.69) |  |
| UA | 356.45±116.99 | 325.69±59 | 0.336 |
| CYSC | 1.37 | 1.15 | 0.339 |
|  | (1.27~1.58) | (1.10~1.67) |  |
| β2-MG | 4756.4 | 2967 | 0.002 |
|  | (4534.40~7451.85) | (2465.94~4626.18) |  |
| Ca | 2.156±0.21 | 2.20±0.17 | 0.503 |
| P | 1.26±0.33 | 1.21±0.27 | 0.628 |
| CK | 47 | 40.5 | 0.661 |
|  | (43.80~84.82) | (29.38~85.99) |  |
| CK-MB | 12 | 15.5 | 0.023 |
|  | (11.29~15.33) | (14.17~24.08) |  |
| LDH | 180 | 189.5 | 0.749 |
|  | (176.45~246.79) | (168.32~239.81) |  |
| LDH1 | 34.5 | 28.5 | 0.379 |
|  | (28.92~47.60) | (25.51~35.99) |  |
| hs-TNI | 6.6 | 3 | 0.001 |
|  | (2.31~90.83) | (1.94~7.27) |  |
| pro-BNP | 633 | 74 | 0.001 |
|  | (446.04~2114.07) | (51.40~85.51) |  |
| RV5 | 1.37±0.57 | 1.11±0.42 | 0.142 |
| SV1 | 0.696±0.561 | 0.58±0.35 | 0.462 |
| QRS | 92 | 88 | 0.237 |
|  | (88.56~104.17) | (85.15~93.79) |  |
| QT | 404 | 389 | 0.474 |
|  | (338.16~421.27) | (376.37~404.49) |  |
| QTs | 441 | 421 | 0.16 |
|  | (379.41~472.21) | (401.93~445.54) |  |
| PR interphase | 151 | 155 | 0.625 |
|  | (118.38~172.76) | (146.60~165.80) |  |
| LVEF | 61 | 62.33 | 0.657 |
|  | (53.78~64.17) | (57.95~66.62) |  |
| EDV | 144.09±44.76 | 122.57±35.05 | 0.097 |
| ESV | 57.21 | 40.15 | 0.155 |
|  | (49.04~70.55) | (37.47~53.48) |  |
| SV | 84.27±30.85 | 76.81±26.64 | 0.41 |
| Myocardial mass (diastolic) | 98.98 | 93.07 | 0.162 |
|  | (93.26~126.54) | (82.22~103.53) |  |
| CO | 6.71±2.65 | 5.68±2.23 | 0.186 |
| CI | 4.10±1.48 | 3.31±1.28 | 0.073 |
| EDV (CF/BSA) | 88.39±26.57 | 71.19±17.85 | 0.022 |
| ESV (CF/BSA) | 34.65 | 21.61 | 0.044 |
|  | (19.16~84.42) | (21.92~31.03) |  |
| SV(CF/BSA) | 52.15±17.42 | 44.92±14.21 | 0.154 |
| Myocardial mass (CF/BSA) | 62.48 | 55.28 | 0.021 |
|  | (58.41~80.65) | (48.42~60.18) |  |
| basal ECV | 39.5 | 31.8 | 0 |
|  | (31.20~68.63) | (29.05~33.88) |  |
| mid ECV | 39.3 | 29.6 | 0 |
|  | (29.68~68.86) | (28.83~33.82) |  |
| apical ECV | 42.25 | 35 | 0.002 |
|  | (29.53~79.16) | (31.18~36.95) |  |
| HGB | 94.69±26.14 | 105.94±25.84 | 0.164 |

**Supplementary Table 3:** Predictive value of ECV in CA

|  | **Cut-off** | **sensitivity** | **specificity** | **AUC** | **95%CI** | **p** | **S.E.** |
| --- | --- | --- | --- | --- | --- | --- | --- |
| basal ECV | 38.35 | 0.929 | 0.8 | 0.911 | 0.819~1 | 0 | 0.047 |
| hs-TNI | 3.15 | 0.929 | 0.36 | 0.647 | 0.464~0.831 | 0.132 | 0.094 |
| LDH1 | 38 | 0.714 | 0.88 | 0.787 | 0.627~0.948 | 0.003 | 0.082 |
| Pro-BNP | 703 | 0.7 | 0.864 | 0.836 | 0.681~0.991 | 0.003 | 0,079 |
| LVEF | 63.885 | 0.2 | 0.591 | 0.414 | 0.192~0.635 | 0.44 | 0.133 |
| B2-MG | 4032.35 | 0.8 | 0.636 | 0.682 | 0.468~0.895 | 0.104 | 0.109 |
| Myocardial mass (CF/BSA) | 75.41 | 0.4 | 0.909 | 0.668 | 0.455~0.881 | 0.133 | 0.079 |

**Supplementary Table 4:** Predictive value of ECV in heart failure

|  | **Cut-off** | **sensitivity** | **specificity** | **AUC** | **95%CI** | **P** | **S.E.** |
| --- | --- | --- | --- | --- | --- | --- | --- |
| ECV base | 37.45 | 0.704 | 0.937 | 0.893 | 0.785～1 | 0 | 0.055 |
| ALB | 32 | 0.481 | 0 | 0.239 | 0.085～.0355 | 0.02 | 0.069 |
| B2-MG | 3395.2 | 0.815 | 0.75 | 0.817 | 0.611～0.917 | 0.04 | 0.78 |
| CK-MB | 12.5 | 0.481 | 0.312 | 0.388 | 0.200～0.577 | 0.26 | 0.096 |
| EDV(CF/BSA) | 86.91 | 0.593 | 0.812 | 0.696 | 0.518～0.874 | 0.049 | 0.091 |
| ESV(CF/BSA) | 33.42 | 0.593 | 0.812 | 0.696 | 0.519～0.873 | 0.049 | 0.09 |
| Myocardial mass (CF/BSA) | 67.43 | 0.444 | 0.937 | 0.708 | 0.541～0.875 | 0.036 | 0.085 |
| hs-TNI | 3.45 | 0.913 | 0.643 | 0.8 | 0.649～0.951 | 0.003 | 0.077 |


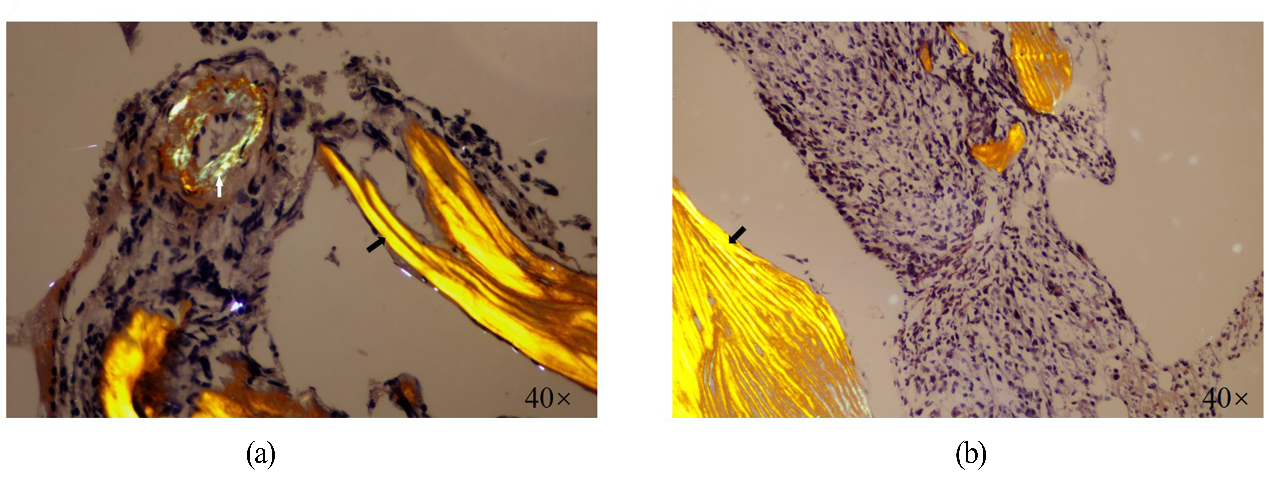


**Supplementary Figure 1**: Fluorescence microscopic photos of amyloidosis **(a)** and non-amyloidosis **(b)** in bone marrow biopsy tissue. **(a)** The white arrow indicates the amyloid deposits in the blood vessel wall of the bone marrow biopsy. The black arrow indicates the trabecular bone. **(b)** No amyloid deposits are detected.
